# Fish Consumption, Long-Chain Omega-3 Polyunsaturated Fatty Acid Intake and Risk of Metabolic Syndrome: A Meta-Analysis

**DOI:** 10.3390/nu7042085

**Published:** 2015-03-24

**Authors:** Yong-Seok Kim, Pengcheng Xun, Ka He

**Affiliations:** 1 Department of Epidemiology and Biostatistics, School of Public Health-Bloomington, Indiana University, Bloomington, IN 47405, USA; E-Mails: harrison72@dumc.or.kr (Y.-S.K.); pxun@indiana.edu (P.X.); 2 Department of Medicine, Graduate School, Dongguk University-Seoul, Seoul, 100-715, Korea

**Keywords:** fish consumption, LCω3PUFA intake, metabolic syndrome, meta-analysis

## Abstract

Fish and long-chain ω-3 polyunsaturated fatty acid (LCω3PUFA) intake in relation to the risk of cardiovascular diseases have been well studied. However, studies that directly link fish consumption or LCω3PUFA intake to the risk of metabolic syndrome (MetS) are sparse and the results are inconsistent. We reviewed literature through December 2014 and used random-effects or fixed-effects models, as appropriate, to pool the associations of fish or LCω3PUFA intake with the risk of MetS. Nine independent cross-sectional samples (seven cross-sectional studies) and three independent prospective cohorts (two prospective cohort studies) were identified as eligible for this meta-analysis. By pooling data from the prospective cohorts (7860 participants and 1671 incident cases), a significant inverse association between fish consumption and incidence of MetS was found. The pooled RR (95% CI) was 0.71 (0.58, 0.87), comparing the highest to the lowest category of fish consumption, and 0.94 (0.90, 0.98) for one serving/week increment. Consistent results were found for LCω3PUFA intake. Non-significant inverse association of fish or LCω3PUFA intake with risk of MetS was found when pooling the cross-sectional studies. By quantitatively summarizing the literature, a modest inverse association between fish or LCω3PUFA intake and risk of MetS cannot be excluded.

## 1. Introduction

Metabolic syndrome (MetS) is considered a major public health problem in the United States as its prevalence increased rapidly in the past two decades. The recent data from the National Health and Nutrition Examination Survey (NHANES) suggest that approximately one third of American adults suffers from this syndrome [1,2]. Therefore, there is an urgent need to control the development of MetS. In this regard, lifestyle interventions, including healthy diet, have been receiving great attention. Accumulated evidence suggests that some food groups such as vegetables, low-fat dairy, and whole-grain products are associated with a lower risk of MetS [3,4,5]. Fish, the primary dietary source of long-chain ω-3 polyunsaturated fatty acids (LCω3PUFA), has also been of great interest because of its potential beneficial effects on the individual components of the MetS [6,7,8,9]. However, studies that directly link fish consumption or LCω3PUFA intake to the risk of MetS are sparse and the results are inconsistent [10,11,12,13,14]. Therefore, this meta-analysis was performed to quantitatively estimate the overall association of fish or LCω3PUFA intake with the risk of MetS based on the published observational epidemiological studies.

## 2. Methods

### 2.1. Data Sources and Searches

This meta-analysis was conducted according to MOOSE (Meta-analysis of Observational Studies in Epidemiology) guidelines in all stages of study design, implementation, and reporting [15]. A systematic literature review was conducted to identify the relevant studies in PubMed through December, 2014 using the terms “fish”, “fish oils”, “seafood”, “animal product”, “omega-3 fatty acid”, “*n*-3 fatty acid”, “metabolic syndrome”, “metabolic syndrome X”, “syndrome X”, and “insulin resistance syndrome.” Additional information was retrieved through Google search and a hand search of the references of relevant articles.

### 2.2. Study Selection

We proposed to search for original research articles including prospective cohort studies, case-control studies, and cross-sectional studies, which were published in English and provided hazards ratio (HR), relative risk (RR) or odds ratio (OR), and the corresponding 95% CIs of MetS in relation to fish or LCω3PUFA intake.

**Figure 1:**
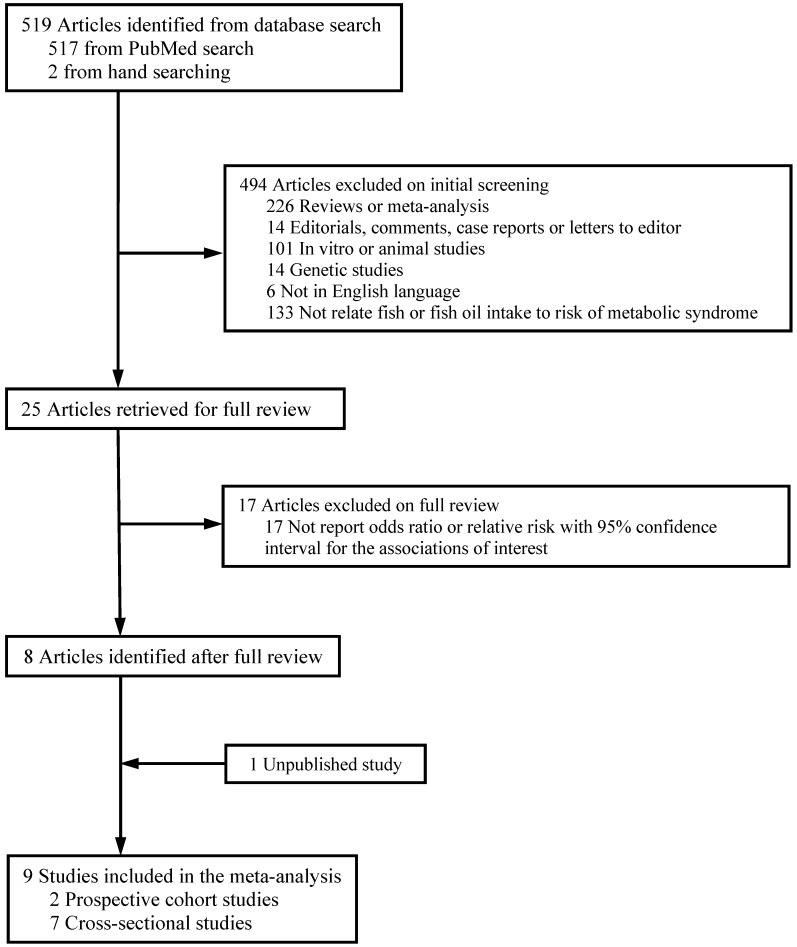
Process of study selection.

As shown in Figure 1, 519 articles were retrieved from PubMed or by hand searching. Of them, 494 articles were excluded by screening due to at least one of the following reasons: (1) 226 were published as reviews; (2) 14 were published as editorials, comments, case reports or letters to editor; (3) 101 were *in vitro* or animal studies; (4) 14 were genetic studies; (5) 6 were not published in English; (6) 133 did not relate fish consumption or LCω3PUFA intake to the risk of MetS. Among the remaining 25 studies, 17 studies were further excluded because they did not provide the data in the form required for the meta-analysis. If a study reported results for male and female participants separately, the study was counted as two independent cohorts in the meta-analysis. An unpublished study with *de novo* results on fish and MetS from the Coronary Artery Risk Development in Young Adults (CARDIA) Study was also included in the present meta-analysis. In the final dataset for the meta-analysis, 7 cross-sectional studies (9 independent samples) and 2 prospective studies (3 independent cohorts) were included.

### 2.3. Data Extraction

The following data were extracted from the original publications: design of the study (cross-sectional or prospective cohort study), the first author’s name, year of publication, study population, country of origin, size of sample, age range or mean age of the participants, proportion of men, duration of follow-up (for prospective cohort studies), method for diet assessment, categories of fish or LCω3PUFA intake, criteria for MetS ascertainment, adjusted covariates, and ORs or RRs with the corresponding 95% CI. All procedures, including the literature search, study selection, and data extraction, were independently performed by two authors (YSK and PX). Any disagreements were solved by group discussion.

### 2.4. Statistical Analysis

In this meta-analysis, OR (derived from cross-sectional studies) or RR (from prospective cohort studies) was used as a measure of effect size, and no eligible cohort studies reported HR. ORs or RRs and 95% CIs were transformed to their natural logarithms (ln) and were used to compute the corresponding standard errors (SEs). Since no significant heterogeneity was observed in most of the pooled analyses, results from the fixed-effects models were reported for the main analysis. The ln (ORs) or ln (RRs) from primary studies was weighted by the inverse of the within-study variance [16].

We also determined the pooled dose-response relationship between fish (one serving/week increment) or LCω3PUFA intake (per 100 mg/day increment) and the risk of MetS in the prospective cohorts. If the primary study did not provide information on linear association, we estimated it based on the categorical analysis using the meta-analysis regression method [17]. For instance, ln (RR) was linearly regressed against its dosage of the exposure using the inverse variance of ln (RR) as the weight for each category. The median intake of fish or LCω3PUFA in each category was derived as the dosage of exposure. Because of the insufficient information, we were not able to assess the dose-response relationship among the cross-sectional studies.

Cochran’s *Q* test was used to determine the heterogeneity among studies statistically [18]. *I*^2^ was computed to quantify the degree of inconsistency across studies [19]; a value of *I*^2^ > 75% was considered as having strong heterogeneity across studies [20]. Publication bias was assessed by the Egger’s regression asymmetry test and Begg’s adjusted rank correlation test. If publication bias did exist, Duval and Tweedie nonparametric “trim and fill” method was used to get the overall estimate [21].

Sensitivity analyses included: (1) investigating the influence of a single study on the combined association by omitting one study at each time in the pooled analysis; and (2) examining whether the overall association was robust depending on modeling by replacing the fixed-effects model with the random-effects model in the pooled analysis.

All analyses were performed using STATA statistical software (Version 13.0, STATA Corp, College Station, TX, USA). *p* ≤ 0.05 was considered statistically significant for all tests.

## 3. Results

### 3.1. Study Characteristics

A total of 9 independent cross-sectional samples and 3 independent prospective cohorts from 9 identified studies (7 cross-sectional studies and 2 prospective cohort studies) were included in the meta-analysis (Table 1). Sample sizes of the included studies varied from 420 to 4941. Four independent samples (including 2 cross-sectional and 2 prospective cohorts) were from Asian populations, and the other studies were conducted in Western countries. Dietary assessment methods included 3- or 4-day food records and a self-administered or interview-based food frequency questionnaire. Among 9 independent cross-sectional samples, 6 reported results on fish consumption, 2 only on LCω3PUFA intake, and 1 sample on both of them. The three independent prospective cohorts reported results on both fish and LCω3PUFA intake. The National Cholesterol Education Program’s Adult Treatment Panel III (ATP III) criterion was used to define MetS in 5 independent cross-sectional samples and one prospective cohort, and the updated ATP-III criteria [22,23] were used in 2 cross-sectional and 2 independent prospective cohorts. In two cross-sectional samples from one cross-sectional study, which were conducted before the ATP III criteria were issued, MetS cases were defined arbitrarily as having two or more of the following four characteristics: serum triglycerides, diastolic blood pressure or fasting glucose in the upper quartile of the distribution, or HDL cholesterol in the lowest quartile. In addition, two studies that were conducted in Asian populations used an ethnicity-specific cut-off point for defining the waist circumference component. For instance, in one study conducted in Iran, waist circumference 95 cm was used as cut-off point for both men and women following the new description of abdominal obesity for Iranian adults [12].

### 3.2. Association between Intake of Fish or LCω3PUFAs and Risk of Metabolic Syndrome

In the pooled analyses of prospective cohorts, a significant inverse association between fish consumption and incidence of MetS was found comparing the highest to the lowest category of exposure (pooled RR: 0.71, 95% CI: 0.58, 0.87). The incidence of MetS reduced by 6% for one serving/week increment in fish consumption (pooled RR: 0.94; 95% CI: 0.90, 0.98). Marginally significant heterogeneity across studies was observed in both categorical analysis (*I*^2^: 60.7%, *p* = 0.08) and dose-response relationship analysis (*I*^2^: 66.3%, *p* = 0.052).

**Figure 2:**
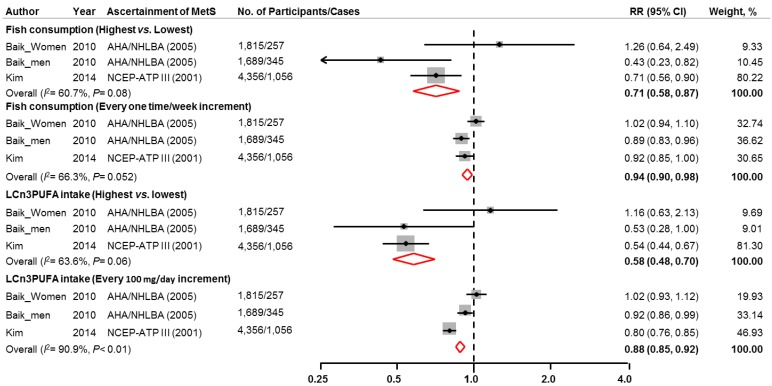
Multivariable adjusted RRs and 95% CIs (horizontal lines) for incidence of metabolic syndrome from prospective cohort studies. The pooled estimates (diamond data markers) were obtained using fixed-effects models. The dots indicate the adjusted RRs by comparing the highest to the lowest category of fish or LCω3PUFA intake or every 1 serving/week increment in fish consumption or 100 mg/day increment in LCω3PUFA intake. The size of the shaded square is proportional to the percent weight of each study. CI: confidence interval; RR: relative risk.

In addition, significant inverse association between LCω3PUFA intake and incidence of MetS was found comparing the highest to the lowest category of exposure (pooled RR: 0.58, 95% CI: 0.48, 0.70) with a marginally significant heterogeneity across the included studies (*I*^2^: 63.6%, *p* = 0.06) (Figure 2). The incidence of MetS was 12% lower with every 100 mg/day increment in LCω3PUFA intake (pooled RR: 0.88; 95% CI: 0.85, 0.92). A significant heterogeneity was found across the studies (*I*^2^: 90.9%, *p* < 0.01).

**Figure 3:**
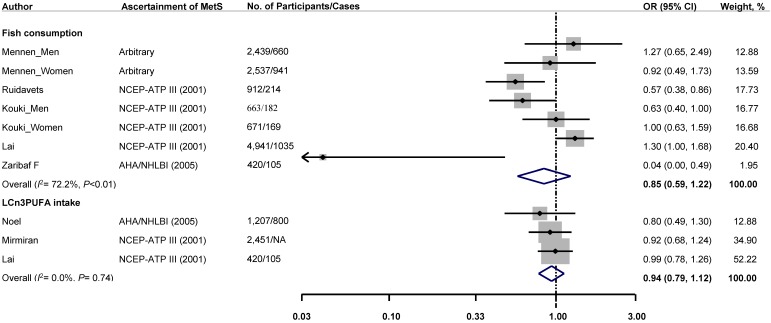
Multivariable adjusted ORs and 95% CIs (horizontal lines) for prevalence of metabolic syndrome from cross-sectional studies. The pooled estimates (diamond data markers) were obtained using a fixed-effects model. The dots indicate the adjusted ORs by comparing the highest to the lowest category of fish or LCω3PUFA intake. The size of the shaded square is proportional to the percent weight of each study. CI: confidence interval; NA, not available; OR: odds ratio.

In the pooled analyses of cross-sectional studies, statistically non-significant inverse associations between fish consumption or LCω3PUFA intake and risk of MetS were observed. The pooled ORs (95% CIs) comparing the highest to the lowest category of exposure were 0.85 (95% CI: 0.59, 1.22) for fish consumption and 0.94 (95% CI: 0.79, 1.12) for LCω3PUFA intake (Figure 3). No significant heterogeneity was found among the included studies for LCω3PUFA intake (*I*^2^: 0.0%, *p* = 0.74), whereas there was significant heterogeneity among studies for fish consumption (*I*^2^: 72.2%, *p* < 0.01). The pooled dose-response relationship in the cross-sectional studies was not assessed due to the insufficient data.

### 3.3. Publication Bias

Egger’s regression asymmetry test indicated no evidence of publication bias for fish consumption (*p* = 0.96 for the categorical analysis and *p* = 0.74 for the dose-response relationship assessment in the prospective cohort studies; *p* = 0.22 for the categorical analysis in the cross-sectional studies). Similarly, there was no evidence of publication bias for LCω3PUFA intake (*p* = 0.56 for the categorical analysis and *p* = 0.17 for the dose-response relationship assessment in the prospective cohort studies; *p* = 0.16 in the cross-sectional studies). Begg’s test confirmed these results.

**Table 1:** Characteristics of included cross-sectional studies and prospective cohort studies on the associations between intakes of fish or LCω3PUFA and risk of metabolic syndrome.

Source	Participants (*n*)	Age (years)	Men (%)	Duration of Follow-Up (Years)	Exposure Assessment	Exposure Categories	Metabolic Syndrome Ascertainment	No. of Cases	Adjusted Variables
Cross-Sectional Studies									
Mennen *et al.* [11], 2000, DESIR study, France	2439	30–64	100	N/A	Self-administered questionnaire	Fish intake	Arbitrary criteria	660	Age, waist-hip ratio and energy intake.
						(portions /week):			
						<2;			
						2–4;			
						>4;			
Mennen *et al.* [11] 2000, DESIR study, France	2537	30–64	0	N/A	Self-administered questionnaire	Fish intake	Arbitrary criteria	941	Age, waist-hip ratio and energy intake.
						<2;			
						2–4;			
						>4;			
Ruidavets *et al.* [14], 2007, MONICA study, France	912	45–64	100	N/A	3-day food record	Fish intake (g/day): Tertiles	NCEP-ATP III	214	Age, center, physical activity, level of education, smoking habits, alcohol intake, drugs for hypertension and dyslipidaemia, energy intake (without alcohol), dieting, and diet quality index.
Noel *et al.* [13], 2010, BPRH Study, USA	1207	45–75	~30 (exact proportion: NA)	N/A	Self-administered questionnaire	*n*-3 PUFA: Quintiles of fat intake as a percentage of total energy	AHA/NHLBI	~800 (exact number: NA)	Age, gender, smoking and alcohol use, physical activity, education, fish oil supplement use, acculturation, total energy, total fat, dietary fiber, lipid-lowering medication use and BMI.
Kouki *et al*. [24], 2011, DR’s EXTRA study, Finland	663	57–78	100	N/A	4-day food record	Fish intake (g/day):	NCEP-ATP III	182	Age, smoking, alcohol consumption, education and VO_2_max.
						<18.5;			
						18.5–59.5;			
						>59.5;			
Kouki *et al.* [24], 2011, DR’s EXTRA study, Finland	671	57–78	0	N/A	4-day food record	Fish intake (g/day):	NCEP-ATP III	169	Age, smoking, alcohol consumption, education and VO_2_max.
						<18.0;			
						18.0–51.0;			
						>51.0;			
Mirmiran *et al.* [12], 2012, TLGS, Iran	2451	19–84	46	N/A	Interviewer-administered questionnaire	Fish oil (EPA + DHA, mg/day):	NCEP-ATP III *	NA	Age, gender, smoking status, physical activity, total energy intake, percentage of energy from carbohydrate, protein, saturated fatty acid, monounsaturated fatty acid, oleic acid, and total fiber.
						≤29;			
						30–66;			
						67–135;			
						≥136.			
Lai *et al.* [10], 2013, NHLBI Family Heart Study, USA	4941	52.1 (mean)	46	N/A	Self-administered questionnaire	Fish intake (times/week):	NCEP-ATP III	1035	Age, gender, race, alcohol intake, smoking, exercise, TV watching, energy intake, multivitamin use, fruits and vegetables intake, and risk group using generalized estimating equations.
						0;			
						1;			
						2;			
						≥3.			
						Dietary *n*-3 PUFA (quintiles, mean (g/day)):			
						Q1: 0.04;			
						Q2: 0.11;			
						Q3: 0.18;			
						Q4: 0.28;			
						Q5: 0.64.			
Zaribaf *et al.* [25], 2014, Iran	420	35.2 (mean)	0	N/A	Self-administered questionnaire	Energy-adjusted fish intake (g/day): Tertiles	AHA/NHLBI	105	Age, energy intake, physical activity, socioeconomic status, medication use, marital and menopausal status, dietary intakes of red meat, whole and refined grains, fruits, vegetables, legume and nuts, dairy products, fiber and oils, BMI.
Prospective Studies									
Baik *et al.* [26], 2010, Korean Genome Epidemiology Study, Korea	1689	40–69	100	4	Self-administered questionnaire	Fish intake (times/week):	AHA/NHLBI *	345	Age, BMI, income, occupation, marital status, education level, smoking status, alcohol intake, physical activity, daily intake of energy, fat, dietary fiber, consumption of red meat, dairy products, sweetened carbonated beverage, use of multivitamin supplements, and baseline report of a physician diagnosis of diabetes or hypertension.
						<1;			
						1–4;			
						5–6;			
						Daily.			
						*n*-3 PUFA (percentile, median (mg)):			
						<10th: 37;			
						10th–50th: 138;			
						50th–90th: 375;			
						>90th: 786 mg.			
Baik *et al.* [26] , 2010, Korean Genome Epidemiology Study, Korea	1815	40–69	0	4	Self-administered questionnaire	Fish intake (times/week):	AHA/NHLBI *	257	Age, BMI, income, occupation, marital status, education level, smoking status, alcohol intake, physical activity, daily intakes of energy, fat, dietary fiber, red meat, dairy products, and sweetened carbonated beverage, use of multivitamin supplements, baseline report of a physician diagnosis of diabetes or hypertension, menopausal status, and postmenopausal hormone use.
						<1;			
						1–4;			
						5–6;			
						Daily.			
						*n*-3 PUFA (percentile, median (mg)):			
						<10th: 29;			
						10th–50th: 125;			
						50th–90th: 360;			
						>90th: 563.			
Kim *et al.* (Under journal review) CARDIA study, USA	4356	18–30	47	25	Interviewer-administered questionnaire	Fish intake:	NCEP-ATP III	1069	Age, gender, ethnicity, study center, education, smoking status, family history of diabetes, physical activity, alcohol consumption, and baseline BMI. Fried fish was also adjusted when non-fried fish was the exposure.
						<1/month;			
						1–3/month;			
						1/week;			
						2–4/week;			
						≥5/week.			
						Fish oil (Quintiles, median (g/day)):			
						Q1: 0.03;			
						Q2: 0.07;			
						Q3: 0.11;			
						Q4: 0.18;			
						Q5: 0.33.			

AHA, American Heart Association; BMI, body mass index; BPRH, Boston Puerto Rican Health; CARDIA, Coronary Artery Risk Development in Young Adults; DESIR, Data from an Epidemiological Study on the Insulin Resistance syndrome; DHA, docosahexaenoic acid; DR’s EXTRA, Dose Responses to EXercise TRAining; EPA, eicosapentaenoic acid; MONICA, MONItoring of trends and determinants in CArdiovascular disease; NA, not available; N/A, not applicable; NCEP-ATP, National Cholesterol Education Program-Adult Treatment Panel; NHLBI, National Heart, Lung, and Blood Institute; PUFA, polyunsaturated fatty acid; TLGS, Tehran Lipid and Glucose Study; VO_2_max, maximal oxygen uptake. * Ethnicity-specific cut-offs for waist circumferences were applied for the definition of metabolic syndrome.

### 3.4. Sensitivity Analysis

To test the robustness of meta-analysis results, we removed 1 original study each time in the pooled analysis. The overall associations in the prospective cohorts were attenuated to marginally significant or non-significant by excluding Kim *et al.*, whereas no single study substantially influenced the pooled association in the cross-sectional studies.

In addition, we replaced the fixed-effects model with the random-effects model in all pooled analyses; our results were essentially unchanged in the pooled cross-sectional samples, but somewhat attenuated in the pooled prospective cohorts (data not shown).

## 4. Discussion

It has been hypothesized that fish or LCω3PUFA intake may be associated with lower risk of MetS [27]. However, data directly linking fish consumption or LCω3PUFA intake to the risk of MetS are sparse. This meta-analysis summarized the up-to-date literature, and findings did not provide strong evidence supporting the hypothesis, though a modest inverse association between fish consumption or LCω3PUFA intake and the risk of MetS cannot be excluded.

Our meta-analysis includes 3 prospective cohorts (~8000 participants) and 7 cross-sectional studies (~16,000 participants). While we acknowledge that the overall sample size may still not be sufficient (e.g., for longitudinal analysis), we believe that findings from this meta-analysis will generate valuable data and stimulate research in this field. Clearly, more longitudinal studies are needed in order to make any solid conclusion. In addition, the limited numbers of included studies may explain how the overall association from prospective cohort studies is influenced by one study, *i.e.*, Kim *et al.* In addition, a few issues merit discussion. First, similar to any other meta-analysis, the inherent limitations of primary studies may have affected our findings. Second, although the individual OR or RR estimate in the primary studies was adjusted for different covariates, the possibility that unmeasured factors or residual confounding biased our findings cannot be completely excluded. For instance, the largest cross-sectional study [10], which contributed up to 52% weight in the meta-analysis, observed that triglyceride levels were higher in the group with the highest fish consumption. This finding is not consistent with the well-established hypotriglyceridemic effect of fish or LCω3PUFA intake from previous studies [28]. The investigators presumed that their findings might be due to confounding by indication. Nevertheless, this paradoxical observation might partially explain the overall non-significant association in the meta-analysis of cross-sectional studies. Third, as discussed in previous publications [8,29,30], fish preparation methods (e.g., frying) and some contaminants in fish may attenuate the potential beneficial effects of fish consumption. Unfortunately, the relevant information was not available in most of the included studies [10,11,12,13,24,25,26]. Fourth, previous intervention studies suggested that LCω3PUFA intake affected individual components of MetS at a relatively high dose [9]. Of note, the average intakes of fish and LCω3PUFAs in the studies included in the meta-analysis were modest, which might result in the modest or non-significant association between fish or LCω3PUFA intake and the risk of MetS. In the prospective cohort study conducted in a Korean population [26], investigators found that fish consumption was associated with lower risk of MetS in men but not in women. The authors assumed that this gender-specific discrepancy was derived from a relatively lower amount of fish consumption in women [26].

The publication bias is always a concern in meta-analysis. Although we found little evidence of publication bias in the present meta-analysis, a potential publication bias resulting from excluding unpublished data or publications in non-English languages cannot be ruled out.

The best approach to evaluate the causality of diet-disease relations is to conduct a long-term, double-blinded, and placebo-controlled randomized trial. However, given the practical and ethical limitations such as the participant’s long-term compliance, it may not be feasible to pursue such a trial on fish consumption and risk of MetS. Thus, we hope findings from this meta-analysis will draw researchers’ attention and call for longitudinal studies of fish consumption and risk of MetS.

## 5. Conclusions

In conclusion, a modest inverse association between fish consumption or LCω3PUFA intake and risk of MetS has been observed when combining available data from prospective cohort studies, but not the cross-sectional studies. More research, especially prospective cohort study, is needed in order to make a solid conclusion and to investigate fish and LCω3PUFA intake in relation to individual components of MetS.
